# Genome-Wide Analysis of the PEBP Gene Family and Functional Characterization of *BcFT-1/2* in *Choy Sum* (*Brassica rapa* subsp. *chinensis* var. *parachinensis*)

**DOI:** 10.3390/ijms27104507

**Published:** 2026-05-18

**Authors:** Baoping Deng, Xiaoyun Xin, Peirong Li, Weihong Wang, Deshuang Zhang, Yangjun Yu, Xiuyun Zhao, Bin Zhang, Fenglan Zhang, Shuancang Yu, Tongbing Su, Shiwei Song

**Affiliations:** 1 College of Horticulture, South China Agricultural University, Guangzhou 510642, China; 2 Beijing Vegetable Research Center (BVRC), Beijing Academy of Agriculture and Forestry Science (BAAFS), Beijing 100097, China; 3 National Engineering Research Center for Vegetables, Beijing 100097, China; 4 Key Laboratory of Biology and Genetic Improvement of Horticultural Crops (North China), Ministry of Agriculture, Beijing 100097, China; 5 Beijing Key Laboratory of Vegetable Germplasm Improvement, Beijing 100097, China

**Keywords:** Brassica rapa, PEBP gene family, plant development, expression analysis, FT, TFL1

## Abstract

Choy Sum (*Brassica rapa* subsp. *chinensis* var. *parachinensis*), also known as flowering Chinese cabbage, is an important stalk vegetable in Asia. However, the unique regulatory mechanism governing its “easy-bolting yet susceptible to premature bolting” trait remains poorly understood. The phosphatidyl ethanolamine-binding protein (PEBP) family serves as a central regulator of bolting, flowering, and growth development in plants. But this gene family has not been systematically identified and studied in Choy Sum yet. Therefore, this study systematically identified and analyzed the members of the PEBP gene family in Choy Sum using bioinformatics, transcriptomics, real-time fluorescence quantification, subcellular localization, and transgenic techniques. A total of 12 BcPEBP genes were identified and categorized into three subfamilies: *FT-like*, *TFL1-like*, and *MFT-like*. Phylogenomic analyses revealed family expansion through whole-genome duplication with strong purifying selection. Most members have highly conserved core motifs and gene structures. Protein sequence alignment showed that *BcFT-2* and *BcTFL-2* underwent non-synonymous mutations at key residues. The analysis of cis-acting elements suggests that the BcPEBP gene may be influenced by complex hormone and light regulatory networks. Expression profiling demonstrated leaf-specific upregulation of *BcFT-1/2* during development and shoot apices-predominant expression of *BcTFL1* genes, and the expression between homologous genes of *BcTFL1-1/3* is more refined. Subcellular localization confirmed dual nuclear and plasma membrane targeting of *BcFT-1/2* proteins. Overexpression of *BcFT-1/2* in transgenic *Arabidopsis* promotes flowering. These findings establish BcPEBP genes as key bolting regulators and provide molecular targets for breeding-improved varieties.

## 1. Introduction

Flowering is a crucial developmental process that marks the transition from the vegetative growth stage to the reproductive growth stage in plants. This process directly determines crop yield and quality. Therefore, its regulatory mechanism has always been a focal point in plant biology research. Choy Sum (*Brassica rapa* subsp. *chinensis* var. *parachinensis*), also known as flowering Chinese cabbage, is a vegetable primarily valued for its tender stalks and stem leaves, which are extensively cultivated and widely consumed across Asia [1]. Vegetative growth and reproductive growth proceed simultaneously, which is significantly different from other cruciferous leafy vegetables, such as Chinese cabbage (*B. rapa*) and Heading cabbage (*B. oleracea*) [2,3] during the development of Choy Sum. Excessively rapid reproductive growth can cause early bolting of Choy Sum, which results in a decrease in the yield and quality of the bolts [4]. Therefore, how to coordinate vegetative growth and reproductive growth is a key aspect of regulating the formation of Choy Sum bolts.

The phosphatidylethanolamine-binding protein (PEBP) gene family regulates various developmental processes such as flowering, plant morphogenesis, and seed germination, and plays a pivotal role in plant adaptive evolution and the formation of agronomic traits [5]. Based on functional and phylogenetic differences, the PEBP gene family can be divided into three subfamilies: *FT-like (FLOWERING LOCUS T-like)*, *TFL1-like (TERMINAL FLOWER 1-like)*, and *MFT-like (MOTHER OF FT AND TFL1-like)*. The functions of its members are both conserved and differentiated [6]. In *A. thaliana*, FT proteins bind to the FD-14-3-3 complex on DNA through their C-terminal tails and form the flowering activation complex (FAC), which then promotes flowering. Its expression level directly determines the timing of flowering [7,8]. TFL1 antagonizes the function of FT by competitively binding to FD proteins, thus inhibiting flowering and maintaining the plant’s vegetative growth and adventitious growth habit [9,10]. Notably, although *BFT* belongs to the *FT-like* subfamily phylogenetically, its homologs in *A. thaliana* and Cotton have been shown to have flowering-inhibiting functions [11,12]. Recent studies have revealed a negative feedback regulation mechanism between *TFL1* and *LFY (LEAFY)* in *A. thaliana*, which differs from traditional competitive inhibition and provides a new perspective for dissecting the complex flowering regulatory network of PEBP proteins [13]. *MFT* mainly participates in the regulation of seed germination and dormancy by regulating the balance between ABA (abscisic acid) and GA (gibberellin) signaling pathways [14]. These research results indicate that the PEBP gene family, through synergistic interactions with its subfamily members or other genes, constitutes the core of the plant flowering regulatory network and plays a crucial role in regulating plant flowering time and growth habits.

The functional conservation of the PEBP gene family has been confirmed in various plants, and its functional differentiation is determined by key amino acid residues, such as Tyr^85^ and Gln^140^ in FT proteins, and His^88^ and Asp^144^ in TFL1 proteins. Moreover, mutations in a single amino acid are sufficient to reverse protein function [15]. Simultaneously, this gene family exhibits significant species specificity in terms of gene number, expression patterns, and regulatory mechanisms. For instance, *A. thaliana* contains six PEBP genes, whereas the gene family is significantly expanded in polyploid Brassica crops such as *B. napus* and *B. juncea* var. tumida [16]. Systematic identification of different Brassica species reveals the presence of 29 BnPEBP genes in B. napus and 21 BjPEBP genes in *B. juncea* var. tumida, among which *BjTFL1* and *BjTFL3* are closely associated with the pre-bolting stage [17,18]. Additionally, 11 ApPEBP genes in *Arabidopsis pumila* have expanded through whole-genome duplication events and exhibit tissue-specific expression and stress response characteristics [19]. These discoveries reveal that the functional differentiation and species-specific evolution of the PEBP gene family in Brassica plants constitute an important biological basis for adapting to diverse environments and meeting agronomic demands.

The PEBP gene family, as one of the key gene families regulating flowering, has not been systematically identified in Choy Sum. Therefore, this study aims to utilize the Choy Sum genome data assembled in our laboratory to identify members of the PEBP gene family and conduct bioinformatics analysis, providing a reference for further exploring the role of this gene family in regulating the bolting and flowering process of Choy Sum.

## 2. Results

### 2.1. Screening and Physicochemical Property Analysis of BcPEBP Family Members

**Figure 1:**
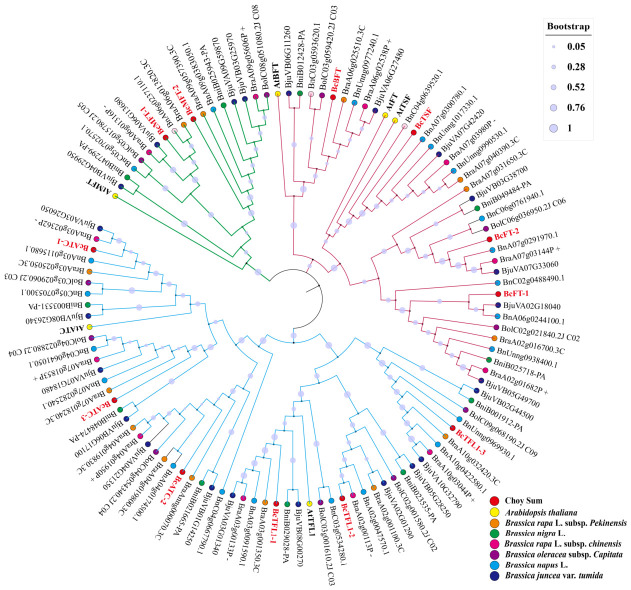
Phylogenetic analysis of PEBP family proteins. The NJ tree contains 114 PEBP proteins, which are derived from Choy Sum, *Arabidopsis*, Chinese cabbage, black mustard, Pak Choi, heading cabbage, semi-winter oilseed, and tuber mustard. The *FT-like*, *TFL-like*, and *MFT-like* subfamilies are marked with red, blue, and green background lines, respectively. BcPEBP members are highlighted with red circles and font, while *A. thaliana* (in bold font), *Brassica rapa* L. subsp. *Pekinensis*, *Brassica nigra* L., *Brassica rapa* L. subsp. *Chinensis*, *Brassica oleracea* L. var. *Capitata*, *Brassica napus* L., and *Brassica juncea* var. *tumida* are represented by yellow, orange, green, pink, purple, cyan, and blue circles, respectively.

In this study, 12 PEBP gene family members were systematically identified in the Choy Sum (*Brassica rapa* subsp. *chinensis* var. *parachinensis*) genome (details in Supplementary Tables S1 and S2). Bioinformatics analysis revealed that all these genes contained the conserved PEBP domain (Pfam: PF01161). Based on phylogenetic analysis results (Figure 1), they were designated as *BcFT-1/2*, *BcTSF*, *BcBFT*, *BcTFL1-1/2/3*, *BcATC-1/2/3*, and *BcMFT-1/2*.

Systematic analysis of the physicochemical properties of the BcPEBP proteins was conducted (Table 1). The results showed that the amino acid residue numbers of this family ranged from 173 to 179 aa, and the molecular weight range was 18.92–20.45 kDa, showing a significant positive correlation with polypeptide length. The isoelectric point (pI) distribution exhibited obvious subfamily specificity: the *FT/ATC-like* subfamily ranged from 7.0 to 7.8, the *MFT-like* subfamily ranged from 7.9 to 8.8, and the *TFL1-like* subfamily reached as high as 8.8–9.6, overall presenting alkaline or weakly alkaline protein characteristics. The grand average of hydropathicity (GRAVY) indices were all negative (−0.338 to −0.112), indicating that BcPEBP genes are mostly hydrophilic proteins. The stability of these physicochemical properties and hydrophilic characteristics provides a structural foundation for their biological functions in plants.

### 2.2. Phylogenetic Tree Analysis of BcPEBPs

To further explore the phylogenetic relationships of BcPEBP genes, we constructed an unrooted phylogenetic tree (Figure 1) using 96 PEBP amino acid sequences from Chinese cabbage (*Brassica rapa* L. subsp. *Pekinensis*, Chiifu_V3.0), black mustard (*Brassica nigra* (L.) W.D.J.Koch, San_1.1), PakChoi (*Brassica rapa* L. subsp. *Chinensis*, PakChoi_V1.0), heading cabbage (*Brassica oleracea* L. var. *Capitata*, JZS_V2.0), semi-winter oilseed (*Brassica napus* L., ZS_2.0), and tuber mustard (*Brassica juncea* var. *tumida* Tsen et Lee, tum_V2.0), identified by ourselves, together with six reported *Arabidopsis* PEBP amino acid sequences and 12 Choy Sum PEBP genes. The results showed that these 114 PEBP proteins from the species were divided into three subfamilies: *FT-like*, *TFL1-like*, and *MFT-like*. Among the BcPEBP gene family members, four (*BcFT-1/2*, *BcTSF*, and *BcBFT*) belonged to the FT-like subfamily, six (*BcTFL1-1/2/3* and *BcATC-1/2/3*) belonged to the TFL1-like subfamily, and two (*BcMFT-1/2*) belonged to the MFT-like subfamily. Phylogenetic analysis highlighted the evolutionary conservation and differentiation of PEBP genes among different species, with Choy Sum retaining functional homologs in all three subfamilies.

### 2.3. Conserved Motif Prediction and Gene Structure Analysis of the BcPEBP Family

**Figure 2:**
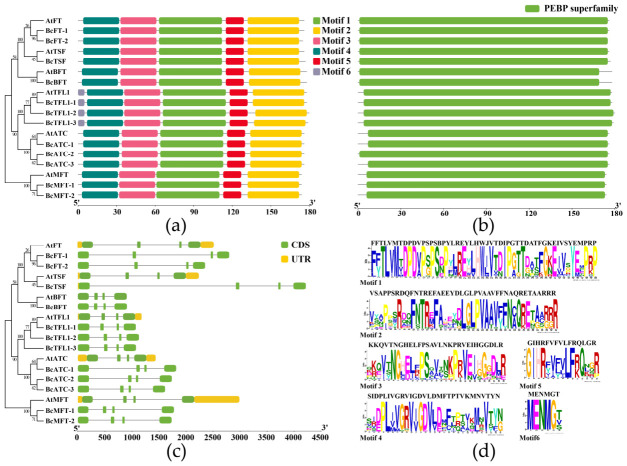
Genetic structural characteristics and conserved motifs of BcPEBPs. (**a**) Conserved motif in BcPEBP proteins. The different colors represent 6 predictive motifs. A phylogenetic tree was constructed on the left using MEGA12.0, which only includes members of *Arabidopsis* and Choy Sum PEBPs. (**b**) Conserved domain of BcPEBP proteins. (**c**) Exon–intron structure of BcPEBP genes. Green boxes represent exons, black lines represent introns, and yellow boxes represent 5′ and 3′ untranslated regions (UTRs). (**d**) Sequence logos of conserved motifs in BcPEBP proteins.

A phylogenetic tree containing only *Arabidopsis* and Choy Sum BcPEBP members was constructed using MEGA12.0 (Figure 2a), showing evolutionary relationships consistent with multi-species systematics (Figure 1). Six conserved motifs were identified in the BcPEBP protein (Figure 2a,d). Except for the *TFL1* gene, containing an additional motif 6, all members contain themes 1–5 arranged in the same sequence. The analysis of conservative domains confirmed the presence of characteristic PEBP domains in all members of the BcPEBP family (Figure 2b). The gene structure analysis showed that members within the same evolutionary branch exhibit similar exon–intron composition patterns. All BcPEBP genes are composed of four exons and three introns (Figure 2c), indicating that the gene structure of BcPEBP is highly conserved during evolution.

### 2.4. Amino Acid Sequence Alignment of the BcPEBPs Family

**Figure 3:**
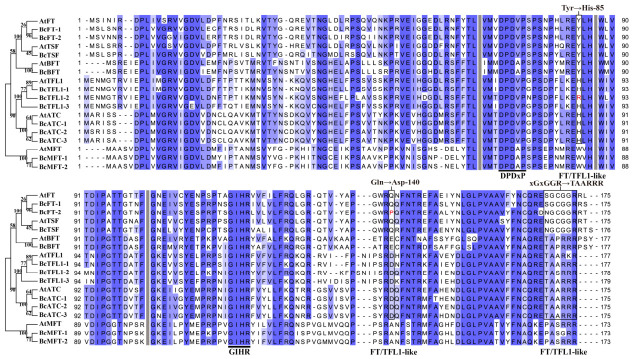
Sequence conservation analysis of Choy Sum and *Arabidopsis* PEBP proteins. Gray vertical lines indicate exon–intron boundaries. Thick black line: DPDxP and GIHR motifs. Black border: key residues and conserved motifs that determine the functional specificity of *FT/TFL1*.The red markings indicate the locations of key amino acid residue mutations in *BcFT-2* and *BcTFL1-2*.

To further investigate the protein structure and functional conservation of BcPEBP genes, protein sequence alignment was performed between *Arabidopsis* and BcPEBP genes (Figure 3). Our results revealed that all BcPEBP proteins harbor two conserved domains, DPDxP (Asp-Pro-Asp-X-Pro) and GIHR (Gly-Ile-His-Arg), which contribute to ligand-binding site formation. In the fourth exon region, FT-like proteins contained xGxGGR, while TFL1-like proteins mostly contained TAARRR. Crucially, our sequence alignment indicates that key amino acid residues, specifically His^88^/Tyr^69^ and Asp^76^/Gln^140^, which control functional differentiation between *FT* and *TFL1* branches, have been altered in some members of Choy Sum. The key amino acid residue at position 88 of *BcTFL1-2* changes from histidine (H) to arginine (R). The key amino acid residue at position 140 of *BcFT-2* changes from glutamine (Q) to proline (P).

### 2.5. Synteny and Selective Pressure Analysis of BcPEBP Family Members in Choy Sum

**Figure 4:**
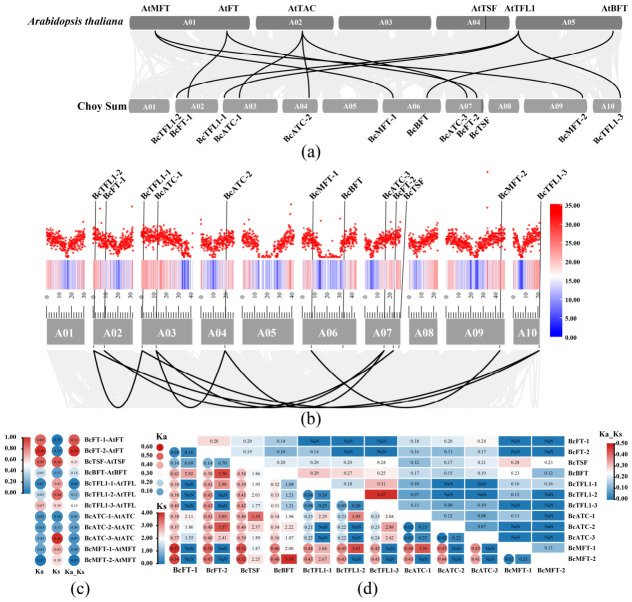
Synteny and selective pressure analysis of the Choy Sum PEBP gene family. (**a**) Interspecific synteny analysis of Choy Sum and *Arabidopsis* PEBP genes. Black lines connect syntenic gene pairs. (**b**) Intraspecific synteny analysis of Choy Sum PEBP genes. Black arcs below connect intraspecific syntenic gene pairs. (**c**,**d**) Selective pressure analysis. (**c**) Ka/Ks ratios of PEBP homologous gene pairs between Choy Sum and *Arabidopsis*. (**d**) Ka/Ks ratios of PEBP paralogous gene pairs within Choy Sum. The numbers inside the circle represent Ka values, the numbers inside the short rectangle represent Ks values, and the numbers inside the long rectangle represent Ka/Ks values. NaN (not a number) represents the synonymous substitution rate Ks ≈ 0 between the gene pairs, making it impossible to calculate the Ka/Ks ratio. It usually originates from very close homologous sequences.

Synteny analysis revealed that 12 Choy Sum PEBP members formed 11 pairs of orthologous syntenic relationships with six Arabidopsis homologous genes, whereas no syntenic relationship was detected between *BcTSF* and *AtTSF*. This absence suggests that a chromosomal rearrangement event may have occurred after species divergence, although alternative explanations (e.g., lineage-specific gene loss) cannot be excluded. The remaining gene pairs retained high levels of macro-synteny (Figure 4a).

Selective pressure analysis showed that the Ka/Ks ratios of all 12 orthologous gene pairs ranged from 0.047 to 0.358, with all values being substantially less than 1, indicative of dominant purifying selection acting on the PEBP gene family between Choy Sum and *Arabidopsis*. Among these pairs, *BcMFT-1/2/AtMFT* exhibited the lowest Ka/Ks values, while *BcFT-1/2/AtFT* showed slightly higher but still substantially sub-family Ka/Ks ratios (Figure 4c).

Intraspecies collinearity analysis identified eight paralogous gene pairs among the 12 BcPEBP members. Segmental duplication events were predominantly detected within the *BcFT*, *BcTFL1*, and *BcATC* subfamilies, whereas *BcTSF* and *BcBFT* existed as single-copy genes (Figure 4b). The Ka/Ks ratios of these paralogous pairs varied from 0.07 to 0.47, suggesting differences in the intensity of purifying selection among duplicated BcPEBP members. Several paralogous pairs, including *BcATC-1/2* and *BcMFT-1/2,* displayed low Ka/Ks values (Ka/Ks < 0.12; Ks ≈ 0.15–0.21), consistent with strong purifying selection. In contrast, *BcTFL1-2/3* presented the highest Ka/Ks ratio (0.47), which may indicate relatively relaxed purifying selection compared with other paralogous pairs; nonetheless, a ratio below 1 remains consistent with the predominant selective constraint (Figure 4d).

### 2.6. Cis-Acting Elements (CAEs) Analysis

**Figure 5:**
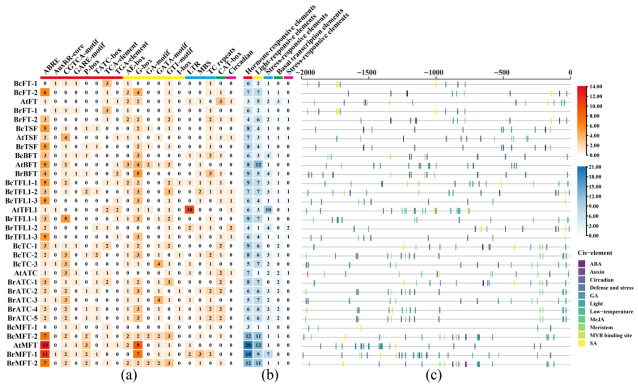
Analysis of cis elements in the PEBP promoter region of Choy Sum, *Arabidopsis*, and Chinese cabbage. (**a**) The color blocks represent the five functional categories of cis elements, and the horizontal lines divide individual genes. The numerical annotation represents the element count of each gene. (**b**) Counting of five functional categories of elements in each PEBP gene promoter. (**c**) The position distribution of each functional component on each promoter in the five functional categories.

Utilizing the PlantCARE online tool, we conducted an analysis of cis-acting elements (CAEs) within the upstream 2000 bp sequences of PEBP gene family members in Choy Sum, *A*. *thaliana*, and *Brassica rapa* (raw data in Supplementary Table S5). The analysis revealed that CAEs related to hormone response in Choy Sum primarily encompass elements involved in abscisic acid response (ABRE, 40), gibberellin response elements (GARE motifs and P-box, 16), and salicylic acid response elements (TCA elements, 9) (Figure 5). Additionally, jasmonic acid methyl ester response elements (CGTCA motifs, 16) were identified, which indicated that Choy Sum PEBP genes may play a central role in flowering regulation and environmental adaptation through interactions with various plant hormones. The main regulatory elements related to abiotic stress included MYB-binding sites (MBS, 7) involved in drought induction, defense, and stress response, TC-rich repeats (11), and low-temperature response elements (LTR, 3). These findings suggest that Choy Sum PEBP genes respond to abiotic stress through multiple pathways, albeit with relatively weaker low-temperature response capabilities. Furthermore, we identified CAEs related to light response (including G-box, GT1 motifs, and AE-box, 65), meristem expression (CAT-box, 9), and circadian regulation (Circadian, 2) (Figure 5a). Based on the number and distribution of promoter CAEs, most members contain response elements for abscisic acid, gibberellin, and jasmonic acid methyl ester, with light response elements accounting for up to 35.5% (Figure 5b,c). This indicates that Choy Sum PEBP genes may play a key role in promoting bolting and flowering of rapeseed and adapting to the abundant sunlight environment in the south by integrating light signaling with multiple hormone-regulatory networks.

### 2.7. Expression Pattern Analysis of the BcPEBPs in Choy Sum Tissues

**Figure 6:**
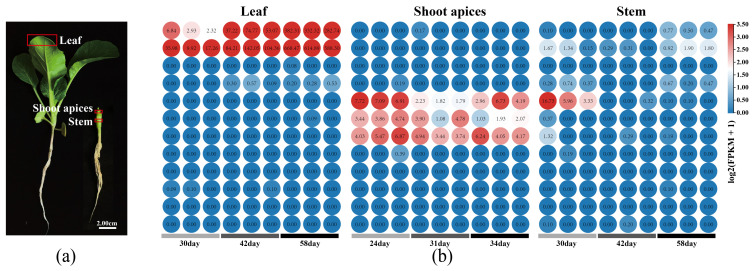
Expression patterns of the PEBP family in different tissues of Choy Sum. (**a**) Shows the growth state of Choy Sum at 30 days and marks the sampling sites corresponding to the RNA-seq. Bar = 2.0 cm. (**b**) Represents three sets of transcriptome data from three tissues: leaves, shoot apices, and stems. Relative expression levels are normalized using Z-score, and the colors in the heatmap indicate expression levels; blue indicates low expression, red indicates high expression, and the numerical value in the circle indicates the number of transcript fragments per kilo bases per million aligned reads (FPKM).

As illustrated in Figure 6b, notable spatiotemporal variations exist in the transcription levels of BcPEBPs within Choy Sum tissues, which suggests that these genes may serve multiple functions during the plant’s growth and development. Specifically, *BcFT-1/2* exhibit high expression levels exclusively in leaves, with their expression upregulated in tandem with reproductive growth. Conversely, *BcTFL1-1/2/3* are predominantly expressed in shoot apices and are downregulated synchronously with reproductive growth. These findings imply that *FT* and *TFL1* genes in Choy Sum are pivotal in maintaining the balance of flower stalk development. Moreover, *BcTFL1-1* displays a distinct expression pattern during the initial stages of stem growth and development, which indicates its potential as a key gene within the *TFL1* family that counteracts *BcFTs* regulation of reproductive growth and development processes. In contrast, *BcBFT* shows low expression levels in both leaves and stems. Additionally, the majority of genes demonstrate nearly negligible expression levels in leaves, shoot apices, and stems (Figure 6a).

### 2.8. Relative Expression Analysis of BcTFL1-1/2/3 in Choy Sum Shoot Apices

**Figure 7:**
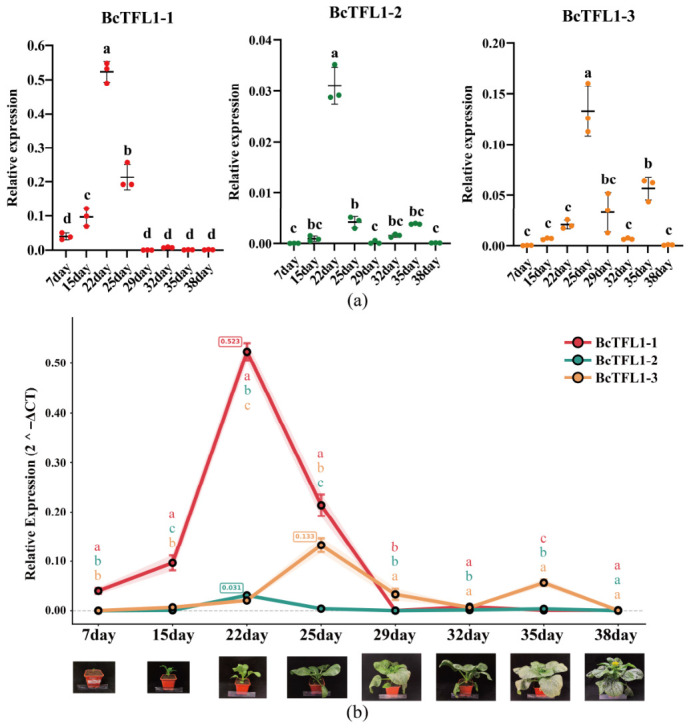
Transcriptional expression analysis of BcTFL1-1/2/3. (**a**) Relative expression levels normalized to day 7. Three biological repeats were used in these assays. Values are the means ± standard deviation. One-way ANOVA followed by Tukey‘s HSD test was performed for multiple comparisons. (**b**) Side-by-side comparison of the three genes at each time point. At each individual time point, one-way ANOVA followed by Tukey‘s HSD test was performed to compare the three genes; different letters indicate significant differences. The following correspond to the growth status of Choy Sum during each sampling period. Different letters (a–d) above the scatter plots indicate significant differences at *p* < 0.05 among time points.

In order to further explore the effect of *BcTFL1* genes on flowering regulation of Choy Sum, based on the characteristics of stem tip-specific expression of the transcriptome, we performed real-time fluorescence quantitative experiments on the stem tip tissue of *BcTFL1-1/2/3* genes on the 7/15/22/25/29/32/35/38 days after sowing (Figure 7a) (raw data in Supplementary Table S7). The results showed that the three copies of the *BcTFL1* family exhibited clear temporal and spatial expression complementarity in shoot apical tissues of Choy Sum. *BcTFL1-1/2/3* showed an overall expression pattern of initially increasing and then decreasing before the bolting stage (32 days). *BcTFL1-1* was significantly highly expressed in the leaf growth period (22 days) and then co-expressed with *BcTFL1-3*, which indicated that *BcTFL1-1* may be the main gene leading flowering regulation of Choy Sum. In addition, *BcTFL1-3* also showed a peak expression at the budding stage (35 days), indicating that *BcTFL1-3* plays a major role in the budding stage (Figure 7b). The relatively low overall expression of *BcTFL1-2* may play an auxiliary regulatory role. This expression differentiation pattern is consistent with the evolutionary flexibility revealed by previous Ka/Ks analysis (Ka/Ks = 0.47 for *BcTFL1-2/3*) (Figure 4d), which provides a molecular basis for understanding the quantitative trait variation in flowering time in Choy Sum.

### 2.9. Heterologous Overexpression of BcFT-1/2 Promotes Flowering in Arabidopsis

**Figure 8:**
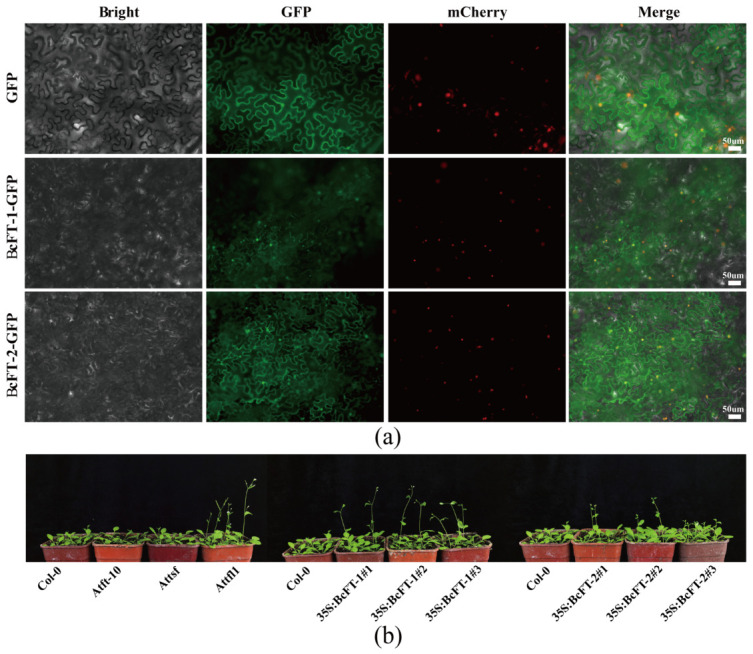
Subcellular localization of BcFT-1/2 proteins and overexpression promoting flowering in *Arabidopsis*. (**a**) Subcellular localization of BcFT-1/2 proteins. BcFT-1/2 were individually inserted into pBI121::35S::GFP, with pBI121::35S::GFP as the control. Bright, bright field; GFP, green fluorescence; mCherry, nuclear marker; Merge, merged image. Bar = 50 μm. (**b**) Overexpression of BcFT-1/2 genes promotes early-flowering phenotypes in *Arabidopsis*. Growth status of wild-type, mutant, and transgenic lines 16 days after transplanting under 24 °C/22 °C, 16 h/8 h environmental conditions. Col-0: *Arabidopsis* wild-type; *Atft-10*: *FT* functional deletion mutant; *Attsf*: *TSF* functional deletion mutant; *Attfl1*: *TFL1* functional deletion mutant.

To elucidate the potential functions of *BcFT-1/2* in the transcriptional regulatory system, we constructed recombinant BcFT-1/2::GFP proteins and transiently expressed them in tobacco (*Nicotiana benthamiana*) leaves. Subcellular localization results showed that BcFT-1::GFP and BcFT-2::GFP fusion proteins co-localized with the nuclear localization marker protein H2B-mCherry in the nucleus, while independent GFP signals were also detected at the cell membrane; the empty vector control showed whole-cell distribution (Figure 8a). To investigate whether BcFT-1/2 possesses flowering-promoting functions, 35S::BcFT-1/2 constructs were introduced into wild-type *A. thaliana*. After kanamycin screening and PCR identification, three T3 generation transgenic plant lines were selected from each construct for seed harvesting and further analysis. Compared with wild-type, *Atft-10*, *Attsf*, and *Attfl1* plants, all transgenic lines exhibited early-flowering phenotypes similar to *Attfl1* (Figure 8b).

## 3. Discussion

In *B. rapa*, flowering time is controlled by multiple environmental and endogenous pathways, including vernalization, photoperiod, thermosensory signaling, GA signaling, and age-dependent regulation, and these cues are ultimately integrated by floral integrators such as *FT* and *SOC1* [20]. In this context, the PEBP family represents a key regulatory layer for bolting and flowering in Choy Sum.

Choy Sum has a weak requirement for low-temperature induction. In South China, year-round supply and rapid crop turnover favor genotypes that bolt and flower quickly. However, the same trait also increases the risk of premature bolting and quality loss. This study shows that the PEBP family is part of the molecular basis of this balance. By combining genome-wide identification, structural analysis, expression profiling, and functional validation, we provided a framework for understanding how Choy Sum maintains rapid flowering while retaining a certain degree of developmental control.

### 3.1. The BcPEBP Family Is Evolutionarily Conserved, but Some Members May Have Diversified During Adaptation

A central point of our evolutionary analysis is that the BcPEBP family is largely conserved, but not static. We identified 12 PEBP genes in Choy Sum (Table 1). This number is higher than that in *Arabidopsis thaliana* (6) but lower than that in polyploid Brassica crops (25 in *Brassica napus*; 23 in *Brassica juncea* var. *tumida*). This result is consistent with the genome ploidy characteristics of Choy Sum as a diploid Brassica crop (2n = 20) and the evolution of gene families in Brassicaceae crops [18], confirming that genome ploidy is a core factor regulating the number of PEBP family members. The three canonical subfamilies, FT-like, TFL1-like, and MFT-like, were all retained. This result suggests that the core flowering functions of the PEBP family were preserved during Brassica evolution [21]. At the same time, conservation does not exclude divergence. Most BcPEBP genes showed clear collinearity with homologs in *Arabidopsis*, and their Ka/Ks values were below 1 (Figure 4a,c). This indicates strong purifying selection, meaning that most duplicated genes have been constrained to maintain their important functions [16,22,23,24]. However, not all genes showed identical syntenic relationships (Figure 4b). These exceptions may reflect local rearrangement, gene loss, or lineage-specific retention after genome duplication [25]. In Choy Sum, such changes may be related to long-term adaptation to warm environments and a reduced dependence on vernalization. Similar evolutionary patterns have been discussed for flowering-time genes in Brassica rapa [19]. This pattern is also consistent with the genome-wide triplication history of B. rapa. Gene retention after WGT (whole-genome triplication) may have increased regulatory complexity and allowed expression and functional divergence among PEBP paralogs, rather than simply expanding gene copy number.

### 3.2. Conserved Structure Supports Shared Function, Whereas Key Residue Changes May Underlie Divergence

The molecular structural characteristics of genes are the basis for the function of realization. In this study, all identified BcPEBP members were characterized as hydrophilic proteins, a trait that likely enhances their cellular solubility and facilitates essential interactions with regulatory partners, such as FD and 14-3-3, during flowering signal transduction [26]. Furthermore, while physicochemical properties, including polypeptide length, molecular weight, and isoelectric point, were highly consistent within each subfamily (Table 1), the significant differences observed between subfamilies provide a clear molecular signature of their functional division. Conserved motif and gene structure analyses further validated the rationality of these evolutionary classifications. Specifically, BcPEBP genes exhibited a highly uniform structure, typically consisting of four exons and three introns (Figure 2). Furthermore, specifically, divergent motifs within the fourth exon—xGxGGR in the FT-like subfamily versus TAARRR in the TFL1-like subfamily (Figure 3)—dictate the binding affinity with transcription factors such as FD and bZIP, establishing the structural foundation for their antagonistic regulation of flowering [27,28,29]. Notably, the non-synonymous mutations detected at key functional residues in BcTFL1-2 and BcFT-2 suggest potential functional attenuation or sub-functionalization, offering precise targets for future site-directed mutagenesis [15].

### 3.3. Promoter Composition and Expression Patterns Are Consistent with the Flowering Habit of Choy Sum

The cis-acting elements of promoters are the “switches” of gene transcription regulation, and their composition determines the spatiotemporal expression characteristics of genes. The total number of cis-acting elements (183) and gene copy number (12) in Chinese cabbage are between those in *Arabidopsis* and Chinese cabbage-related research data (Figure 1 and Figure 5), forming a “transitional” regulatory network. Among them, the proportion of light-responsive elements is as high as 35.5%, while the number of low-temperature-responsive elements is the smallest, at only three. This feature is consistent with the cultivation biology characteristics of Chinese cabbage as a warm and long-day vegetable in southern China [9]. Hormone response elements dominate (47%), significantly increasing in ABA (40) and GA (16) response elements, endowing Chinese cabbage with strong environmental adaptability and regulatory potential [4,30]. It is worth noting that although Chinese cabbage belongs to the Brassica genus, its regulatory network is more “streamlined and efficient” (accounting for only 77% of the total number of components in Chinese cabbage), and the key genes, such as *MFT,* show significant copy-to-copy functional differentiation (the regulatory elements of *BcMFT-1* are greatly simplified to five). In addition, the thermal stress regulation mechanism of *FT* in radish may differ from that in *Arabidopsis*, indicating that the cis-regulatory structure of the BcPEBP gene needs to be interpreted in the unique biological context of Brassica. This may explain why Choy Sum exhibits a regulatory pattern that adapts to rapid bolting under subtropical production conditions. By analyzing the organizational expression patterns, we further identified the core functional genes of the PEBP family. *BcFT-1/2* is consistently expressed in growing leaves (Figure 6), and this expression pattern is highly consistent with *Arabidopsis* [31]. In B. rapa, environmental and developmental signals are mainly perceived in the leaves. After *FT* transcription in the leaves, its protein migrates to the apical meristem of the stem, interacts with FD, and activates the process of flower development. Therefore, the preferential expression of *BcFT-1/2* in leaves strongly supports their core role in the induction of systemic flowering in Chinese cabbage. In addition, previous studies have revealed an antagonistic inhibitory relationship between *TFL1* and *FT* [32,33]. The transcriptome and real-time fluorescence quantitative data in this study (Figure 6 and Figure 7) further indicate that *BcTFL1-1* and *BcTFL1-3* may play an important role in the synergistic regulation of flowering in Chinese cabbage, with *BcTFL1-1* mainly inhibiting flowering and *BcTFL1-3* having a more precise effect on regulation. The regulation balance among these components can ensure timely bolting of Chinese cabbage and reduce quality decline caused by premature flowering. This coordination mechanism is an important molecular strategy for Choy Sum to adapt to intensive commercial demand.

### 3.4. BcFT-1 and BcFT-2 Both Promote Flowering

Subcellular localization and heterologous overexpression experiments directly verified the flowering regulatory functions of *BcFT-1/2*. Both proteins were localized within the nucleus (Figure 8a), consistent with the localization characteristics of *FT* homologous genes, such as *BjFT1* and *SP5G* [17,34]. This indicates that BcFT-1/2 may have dual functions of transcriptional regulation and extracellular signal transduction. Heterologous overexpression in *A. thaliana* demonstrated that both *BcFT-1* and *BcFT-2* are capable of promoting flowering (Figure 8b), confirming their conserved flowering-promoting functions. This finding is consistent with previous reports in *B. rapa* [35]. Additionally, through integrated analysis of protein structure, promoter motifs, and transcript levels of *BcFT-1/2*, we found that *BcFT-2* possesses more abundant promoter cis-elements and exhibits higher transcript levels than *BcFT-1* in Choy Sum. Moreover, a mutation at the key amino acid site (Gln^140^) of *BcFT-2* may be associated with reduced regulatory activity [29]. It is speculated that Choy Sum compensates for the weakened protein function by increasing the transcription level or endows *BcFT-2* with secondary functions to synergistically regulate the flowering process. This finding not only clarifies the functional division of *BcFT-1/2* but also provides insights into the “functional compensation” regulatory strategy formed by Choy Sum during long-term domestication. It is worth noting that heterologous overexpression may not fully reflect the natural function of *BcFT-1/2* in the original plant due to differences in promoters and protein interaction networks. It is recommended to use stable transformation or gene editing in this species for validation in the future.

## 4. Materials and Methods

### 4.1. Genome Data Sources

This study used published Choy Sum genome data (oulv 701, a highly inland line developed by the Guangzhou Institute of Agricultural Sciences, Guangzhou, Guangdong, China) for experimental analysis (the genome file can be downloaded from the National Gene Bank database (CNGBdb) under the biological project CNP0001211) [36], and a laboratory independently assembled Choy Sum genome data (latest cooking 80-day variety, self-pollinated and purified for more than 10 generations) used by the Cabbage Research Group of the Beijing Academy of Agriculture and Forestry Sciences. Third-generation sequencing technology was used to validate the results. *Arabidopsis thaliana* (TAIR10.1) genome information was obtained from The *Arabidopsis* Information Resource database (https://www.arabidopsis.org/ (accessed on 8 January 2026)). Genome information for Chinese cabbage (*Brassica rapa* L. subsp. *Pekinensis*, Chiifu_V3.0), black mustard (*Brassica nigra* (L.) W.D.J.Koch, San_1.1), Pak Choi (*Brassica rapa* L. subsp. *Chinensis*, PakChoi_V1.0), heading cabbage (*Brassica oleracea* L. var. *Capitata*, JZS_V2.0), Semi-winter oilseed (*Brassica napus* L., ZS_2.0), and tuber mustard (*Brassica juncea* var. tumida Tsen et Lee, tum_V2.0) was retrieved from the Brassica Database (BRAD, http://brassicadb.cn/ (accessed on 8 Jan 2026)). It contains multiple Brassica genomes for phylogenetic collinearity comparison to identify species-specific expansion/contraction and gene replication events.

### 4.2. Identification and Physicochemical Property Analysis

The 6 reported *Arabidopsis* PEBP gene amino acid sequences were used as references to perform BlastP comparison (E-value ≤ 1 × 10^−5^) with Choy Sum protein sequences using the TBtools-II (V2.420) software [37]. Meanwhile, the PEBP-conserved domain (PF01161) was downloaded from the Pfam database (http://pfam.xfam.org/) [38], and the Sample HMM Search tool in the TBtools-II software was employed to search Choy Sum and *Arabidopsis* protein data. The intersection of results from these two methods was taken as the final identified members of the Choy Sum PEBP gene family. The ExPASy online tool (https://web.expasy.org/protparam/ (accessed on 15 February 2026)) was used to predict the physicochemical properties of the identified PEBP proteins, including molecular weight (MW), theoretical isoelectric point (pI), and grand average of hydropathicity (GRAVY) [39].

### 4.3. Phylogenetic Tree Construction

The PEBP gene family members of Chinese cabbage, Pak Choi, heading cabbage, black mustard, semi-winter oilseed, and tuber mustard were identified according to the methods described in Section 2.2. Amino acid sequences of PEBP gene family members from each species were extracted (all protein sequences in Supplementary Table S4), and the MEGA 12.0 software (http://www.megasoftware.net/ (accessed on 10 May 2026)) was used to construct phylogenetic trees using neighbor joining [40]. The parameters were established as follows: bootstrap method, 1000 replicates; model/method: Poisson correction model; uniform rates; and pairwise deletion option. The completed trees were beautified using the iTOL v7 online software (https://itol.embl.de/ (accessed on 10 May 2026)).

### 4.4. Conserved Motif Prediction and Gene Structure Analysis

The MEME suite 5.5.9 online tool (http://meme-suite.org) was employed to analyze conserved motifs in *Arabidopsis* and Choy Sum PEBP protein sequences using the multiple expectation maximization model, with the motif width set to 6–50 aa and the maximum motif number set to 6; other parameters were set to default. The sequences were submitted to the NCBI CDD database to verify the existence of conserved PEBP domains. Based on the whole-genome GFF annotation files of *Arabidopsis* and Choy Sum, the Gene Structure Display Server 2.0 (GSDS 2.0, http://gsds.cbi.pku.edu.cn) was used to analyze the exon and intron distribution characteristics of PEBP genes. The above analysis results were visualized using the TBtools-II (V2.420) software.

### 4.5. Protein Sequence Alignment

The MEGA 12.0 software was used to perform multiple sequence alignment of protein sequences from Choy Sum and *Arabidopsis* PEBP gene family members, employing the MUSCLE algorithm with the following parameter settings: a gap-opening penalty of −2.9, a gap extension penalty of 0, and maximum iterations of 16. After visually inspecting for misaligned residues at both ends and obvious mismatches (such as frameshift insertions/deletions in the hypervariable region) produced by MUSCLE, the Jalview 2.11.5.1 (https://www.jalview.org/) software was used for visualization [41].

### 4.6. Synteny and Selective Pressure Analysis

The One Step MCScanX-Super Fast tool in the TBtools-II (V2.420) software was used to predict homologous genes between Choy Sum and *Arabidopsis* species, as well as within the Choy Sum genome. BlastP comparison (E-value ≤ 1 × 10^−5^) was performed between Choy Sum and *Arabidopsis* genomes, and gene duplicate pairs in the PEBP gene family were extracted to determine orthologous genes between the two species (drawing data in Supplementary Tables S9 and S10). BcPEBP protein sequences were subjected to BlastP comparison (E-value ≤ 1 × 10^−5^) within the Choy Sum genome to screen for paralogous BcPEBP genes within the genome. The Dual Synteny Plot and Advanced Circos visualization tools in this software were used to display the syntenic relationships of orthologous genes between Choy Sum and *Arabidopsis*, and paralogous genes within the Choy Sum genome, respectively. The Simple Ka/Ks Calculator (NG) tool in this software was used to calculate the selective pressure on PEBP Gene duplicate pairs.

### 4.7. Promoter Cis-Acting Element Analysis

The TBtools-II (V2.420) software was used to extract 2000 bp sequences upstream of the start codon of PEBP genes from *Arabidopsis* (TAIR10.1), Chinese cabbage (Chiifu_V3.0), and Choy Sum (promoter sequence in Supplementary Table S3). The PlantCARE online tool (http://bioinformatics.psb.ugent.be/webtools/plantcare/html/ (accessed on 20 February 2026)) was employed to predict cis-acting elements in this region [42]. The prediction results were manually verified, and elements lacking biological functional validation, belonging to transposons, enhancers, and non-plant-specific elements, were excluded. The filtered effective cis-acting elements were visualized using R (v4.5.1).

### 4.8. Analysis of BcPEBP Gene Expression in Different Tissues of Choy Sum

Total RNA was extracted from the leaves, stems, and shoot apices of Choy Sum, with three biological replicates at each stage. RNA-seq extract, library preparation, and sequencing were performed on an Illumina NovaSeq platform by Wuhan IGeneBook Biotechnology Co., Ltd., Wuhan, China. Raw reads were quality-filtered using fastp (v0.11.5) and aligned to the *B. rapa* reference genome (v3.0) using HISAT2 (v2.0.1-beta). To measure gene expression levels, the total number of FPKM values for each gene was calculated based on the length of the gene and the count of reads mapping to that gene. The data for each row were normalized and plotted according to the average FPKM value for each gene. The transcriptome data are part of an ongoing study and have not been deposited in a public database. Expression data for the PEBP gene family are provided in Supplementary Table S6.

### 4.9. Total RNA Isolation, cDNA Synthesis, and Real-Time Quantitative PCR Analysis

The samples were selected from the shoot apices of Choy Sum grown for 7/15/22/25/29/32/35/38 days, excluding stamen tissue, and were mixed with three independent strains for three biological replicates of the experiment. Total RNA was extracted from the shoot apices of Choy Sum using the Eastep Super Total RNA Extraction Kit (Promega, Shanghai, China) and using the YEASEN reverse transcription kit (Hifair)^®^ (Yeasen Biotechnology Co., Ltd., Shanghai, China). Third 1st Strand cDNA SynthesisSuperMix for qPCR was used to synthesize the first strand of cDNA, using the 3-phosphoglyceraldehyde dehydrogenase gene (*GAPDH*) as an internal reference gene. All specific primers were as follows: BcTFL1-1-forward: 5′-AGCACCTGCATTGGATAGTAACA-3′; BcTFL1-1-reverse: 5′-CTCTCGAAGGAATGTTTGGGAAGA-3′; BcTFL1-2-forward: 5′-GCCATGAGCTTTTCCCTTCCTT-3′; BcTFL1-2-reverse: 5′-TCCCTATATTTGGCCTTGGGAACT-3′; BcTFL1-3-forward: 5′-CTGCCAAAGCCAAACATAGGGA-3′; BcTFL1-3-reverse: 5′-CGCGAGTGTTGAACTGATCC-3′; BcGAPDH-forward: 5′-CAGGTTTGGAATTGTCGAGG-3′; and BcGAPDH-reverse: 5′-GAGCTGTGGAAGCACCTTTC-3′. Each sample underwent three technical repetitions. According to the method of Reference 2 (-Delta Delta C (T)), the method of 2 (-Delta C (T)) was used for this gene expression analysis [43]. GraphPad Prism 10.6.0 was used to generate the map.

### 4.10. Subcellular Localization of BcFT-1/2 Proteins

To clarify the subcellular localization characteristics of BcFT-1/2 proteins, their complete coding sequences were inserted into the pBI121::35S::GFP expression vector to construct recombinant fusion expression vectors pBI121::35S::BcFT-1/2::GFP. These recombinant vectors and the empty vector control (pBI121::35S::GFP) were transformed into Agrobacterium tumefaciens strain GV3101 by the heat-shock method and then transiently expressed in tobacco leaves by infiltration. The infiltrated tobacco plants were cultured for 2–3 days, and a Zeiss upright fluorescence microscope (Carl Zeiss, Oberkochen, Germany) was used to observe and analyze the fluorescence signal distribution of BcFT-1/2::GFP fusion proteins to determine their subcellular localization (raw data in Supplementary Table S8).

### 4.11. Arabidopsis Genetic Transformation

The floral dip method was employed to infect *Arabidopsis* inflorescences with Agrobacterium carrying recombinant plasmids (pBI121:35S::BcFT-1/2::GFP) to achieve genetic transformation of *BcFT-1/2* genes [44]. Transformed plants were cultured at 24 °C/22 °C under a 16 h light/8 h dark photoperiod until bolting and seed setting. After harvesting mature seeds, resistance screening was performed using 50 mg/mL of kanamycin, and green healthy seedlings growing normally on a resistant medium were selected as candidate transgenic plants. The candidate plants were molecularly detected by rapid PCR technology to verify whether the *BcFT-1/2* genes were integrated into the *Arabidopsis* genome. Positive transgenic plants were continuously self-pollinated to the T3 generation to obtain homozygous transgenic lines with consistent genetic backgrounds, using three independent transgenic Arabidopsis lines, and phenotypic variation characteristic of *BcFT-1/2* overexpression lines was observed and analyzed. The *Arabidopsis Atft-10/Attsf/Attfl1* mutant was purchased from Arashare Company (https://www.arashare.cn).

## 5. Conclusions

This study identified and analyzed 12 members of the BcPEBP family, which were divided into three subfamilies based on systematic evolution—*FT-like*, *TFL1-like*, and *MFT-like*—and summarized the characteristics of each subfamily. The BcPEBP gene family has undergone strong purifying selection during evolution, and members of each family share similar conserved structural features. The analysis of cis-acting elements suggests that the BcPEBP gene may be influenced by a complex network of hormones and light regulation. The transcriptome and real-time fluorescence quantification results showed that *BcFT* and *BcTFL1* were highly expressed in leaves and stem tips, respectively, and the homologous genes of *BcTFL1* exhibited significant spatiotemporal specific expression, suggesting that these genes may be involved in regulating the complex flowering process of Choy Sum. Subcellular localization confirmed that *BcFT-1/2* is located in the nucleus and cell membrane. In transgenic *Arabidopsis*, both *BcFT-1* and *BcFT-2* significantly promote flowering.

In conclusion, our results show that the BcPEBP family combines evolutionary conservation with limited but meaningful divergence. This combination appears well-suited to the biology of Choy Sum. Conserved family structure maintains the core flowering network, while divergence among specific paralogs may support rapid flowering under local cultivation conditions. These findings extend the current understanding of flowering regulation in Brassica rapa and provide candidate genes for breeding cultivars with improved resistance to premature bolting.

## Figures and Tables

**Table 1:** Information on Choy Sum PEBP gene family members.

Gene Name	*A. thaliana* (TAIR10.1)	B. rapa (Chiifu_V3.0)	Protein Length (aa)	MW (kDa)	pI	GRAVY
BcFT-1	AT1G65480	BraA02g016700.3C	175	19.81	7.75	−0.338
BcFT-2		BraA07g031650.3C	175	19.77	7.82	−0.338
BcTSF	AT4G20370	BraA07g040390.3C	176	19.95	7.72	−0.293
BcBFT	AT5G62040	BraA06g025510.3C	177	20.15	9.51	−0.237
BcTFL1-1	AT5G03840	BraA03g001350.3C	177	20.11	9.56	−0.159
BcTFL1-2		BraA02g001100.3C	179	20.45	8.79	−0.162
BcTFL1-3		BraA10g032420.3C	178	20.40	9.51	−0.317
BcATC-1	AT2G27550	BraA03g025050.3C	175	19.82	7.01	−0.232
BcATC-2		BraAnng000070.3C	175	19.89	7.76	−0.273
BcATC-3		BraA07g018240.3C	175	19.78	7.01	−0.236
BcMFT-1	AT1G18100	BraA06g013820.3C	173	18.92	7.87	−0.112
BcMFT-2		BraA09g057390.3C	173	19.07	8.79	−0.174

MW, molecular weight; PI, isoelectric point; GRAVY, grand average of hydropathicity.

## Data Availability

The original contributions presented in this study are included in this article/Supplementary Information. Further inquiries can be directed to the corresponding authors.

## Supplementary Materials

The following supporting information can be downloaded at https://www.mdpi.com/article/10.3390/ijms27104507/s1.
